# Determinants of interspecific variation in season length of perennial herbs

**DOI:** 10.1093/aob/mcad088

**Published:** 2023-07-03

**Authors:** Tomáš Koubek, Tereza Mašková, Tomáš Herben

**Affiliations:** Department of Botany, Faculty of Science, Charles University, Benátská 2, CZ-128 01, Praha 2, Czech Republic; Department of Botany, Faculty of Science, Charles University, Benátská 2, CZ-128 01, Praha 2, Czech Republic; Ecology and Conservation Biology, Institute of Plant Sciences, University of Regensburg, Regensburg 93053, Germany; Department of Botany, Faculty of Science, Charles University, Benátská 2, CZ-128 01, Praha 2, Czech Republic; Institute of Botany, Academy of Science of the Czech Republic, CZ-252 43, Průhonice, Czech Republic

**Keywords:** Season length, growing season, growth phenology, leaf dry matter content, perennial plants, specific leaf area

## Abstract

**Background and aims:**

Perennial plants in seasonal climates need to optimize their carbon balance by adjusting their active season length to avoid risks of tissue loss under adverse conditions. As season length is determined by two processes, namely spring growth and senescence, it is likely to vary in response to several potentially contrasting selective forces. Here we aim to disentangle the cascade of ecological determinants of interspecific differences in season length.

**Methods:**

We measured size trajectories in 231 species in a botanical garden. We examined correlations between their spring and autumn size changes and determined how they make up season length. We used structural equation models (SEMs) to determine how niche parameters and species traits combine in their effect on species-specific season length.

**Key results:**

Interspecific differences in season length were mainly controlled by senescence, while spring growth was highly synchronized across species. SEMs showed that niche parameters (light and moisture) had stronger, and often trait-independent, effects compared to species traits. Several niche (light) and trait variables (plant height, clonal spreading) had opposing effects on spring growth and senescence.

**Conclusions:**

The findings indicate different drivers and potential risks in growth and senescence. The strong role of niche-based predictors implies that shifts in season length due to global change are likely to differ among habitats and will not be uniform across the whole flora.

## INTRODUCTION

Perennial plants in seasonal climates have to cope with changes in temperature, precipitation and light regimes, which lead to pronounced seasonality of growth of most plant species (Falge *et al.*, 2002). A perennial plant, with its long-living below-ground structures and annual above-ground shoots and leaves, has limited time to build light-capturing structures, accumulate sufficient resources and store them below ground before the end of the season. Good timing of phenological events thus permits individual species to adjust the length of their active season to maximize carbon, water and nutrient acquisition and to avoid losses caused by frost, drought and light shortage due to competition (Lechowicz, 1995; Augspurger *et al.*, 2005; Kooyers, 2015).

The length of the active season of each species (further referred to as season length) is determined by its start and end. While in most temperate habitats the beginning and the end of the time when plants are able to grow and assimilate are constrained by one factor, i.e. frost (Augspurger, 2013), processes at the beginning and the end of the growing season are not a simple reversal of each other (Huang *et al.*, 2018; Mašková *et al.*, 2022). Plant growth and senescence are controlled by different signals and respond to different selective forces. Consequently, to understand species’ resource economies and the evolutionary pressures that influence them, it is necessary to investigate drivers of both spring growth and senescence phenology separately and use this information to determine how they combine into the overall season length.

In spring, plant growth is generally limited by unpredictability of late frosts, which can damage or kill developing tissues and thus can seriously damage a plant’s performance for the whole season (Augspurger, 2013; Lee *et al.*, 2022). This leads to an overall synchronization of spring growth (Huang *et al.*, 2019). Nevertheless, selection for phenological divergence, due to competition either for light or for pollinators, is responsible for species starting their growth in spite of such potential late frosts (Lechowicz, 1995). In addition, growth in dry habitats can be pushed to the beginning of the season because of a lack of moisture in summer (Castillioni *et al.*, 2022; Lu *et al.*, 2023); similarly, a shortage of light later in the season may be limiting for growth in productive grasslands and forests. Such forces select some species to grow early (Whigham, 2004; Augspurger *et al.*, 2005; Augspurger and Salk, 2017) and call for specific phenotypes and patterns of functional traits, such as traits of the leaf economy spectrum (Sporbert *et al.*, 2022) or organ preformation before winter (Schnablová *et al.*, 2021).

Tradeoffs in the autumn are different as plants risk much less by losing their above-ground organs at the end of their life but are also under conditions of much less light available for photosynthesis. Nutrient resorption from shoots is the main problem that plants must deal with in autumn (Killingbeck, 1996). Nutrient resorption, shoot senescence and biomass die-off often take place in a programmed way governed by photoperiod, night temperatures or both (Delpierre *et al.*, 2009; Lang *et al.*, 2019). Again, there are strong interspecific differences due to avoidance of shade and drought that may force individual species to extend their growing season until late autumn, with some species being able to accumulate a significant portion of their carbon budgets after tree leaf drop (Heberling *et al.*, 2019). In contrast to leaf abscission in trees, senescence of above-ground shoots in herbs has generally been much less studied and hence is much less understood (Gallinat *et al.*, 2015), in spite of its potential effect on ecosystem productivity (e.g. Wu *et al.*, 2013) and higher sensitivity to changes in temperature compared to spring growth (Fu *et al.*, 2018). Interspecific differences in senescence patterns are again associated with differences in functional traits, such as plant height, low specific leaf area (SLA) and high leaf dry matter content (LDMC) in late senescing species (Bucher and Römermann, 2021; Mašková *et al.*, 2022; Sporbert *et al.*, 2022).

Interspecific differences at both the beginning and the end of the season are thus likely to be associated with similar functional traits, which in turn are determined by species niches (i.e. the environments where these species typically occur). In this paper, we aim to disentangle the cascade of these determinants of interspecific differences in season length and its components, namely spring growth and autumn senescence. While most of the current research on plant phenology (Menzel and Fabian, 1998; Cleland *et al.*, 2007; Wolkovich *et al.*, 2022) addresses intraspecific phenological shifts due to changing climate, here we are interested in interspecific differences in the timing of growth and senescence and their drivers. Our primary aim was thus to compare across species in order to identify how differences in their niches affect species-specific season lengths. First, we investigated whether season length was determined more by differences in spring or autumn phenology and how these variables were related. Second, we aimed to determine whether events of the spring and autumn phenology were linked to the same or different traits of the plant functional spectrum, namely height, clonality and photosynthesis-related traits. Third, we explored how interspecific differences in the timing of spring and autumn events depended on species niches (environmental conditions where these species typically occur). This allowed us to determine whether habitat-specific selective forces affect spring and autumn events in a similar or different fashion.

To this end, we assembled a dataset of 231 species for which we measured both spring growth and senescence phenology in a botanical garden. This provided a direct comparison of season length (i.e. its start and end) across a number of species under identical climatic conditions. Moreover, we were able to identify the species from the very beginning of their growth and therefore go beyond the common recording of simple phenological events such as leaf-out and flowering (see e.g. Denny *et al.*, 2014). Since growth and competition of individuals are governed by size changes of their assimilative structures, we think that vegetative growth data can be ecologically more relevant. Therefore, we measured size trajectories of individual shoots to determine the timing and speed of both spring growth (improving on methods of Huang *et al.*, 2018) and of the senescence phases (Mašková *et al.*, 2022). We then examined correlations between them and sought their predictors among traits of the plant functional spectrum and their niche parameters. To describe the plant functional spectrum, we chose two traits at the plant level, namely height (as a proxy for the ability to compete for light) and lateral spread (as a proxy for the ability to occupy space in the horizontal dimension) and two key traits of the leaf economy spectrum (SLA and LDMC). Finally, we used structural equation models (SEMs) to draw a synthetic picture of the interrelationships of all these variables in their effect on interspecific differences in season length.

## MATERIALS AND METHODS

### Field data collection

The species selection was largely identical to that of Mašková *et al.* (2022). We chose perennial herbaceous species from Central European angiosperm flora to cover sufficiently both functional and phylogenetic diversity. The species were first selected from the permanent collection of the Botanical Garden of Charles University in Prague (50.071°N, 14.420°E). This resulted in 200 species that grew in four garden parts, which were then complemented by 31 species collected in the wild or obtained from a commercial supplier (Planta Naturalis, Markvartice, Czech Republic) in the year before the measurements began. These additional species were chosen to cover either common species or species from underrepresented families missing in the garden. This resulted in 231 species altogether. The species nomenclature follows the Czech determination key (Kaplan and Danihelka, 2019)

The plants in the Botanical Garden collection grew in semi-natural conditions (forest species under trees, grassland species in open places), separated by a sufficient distance to reduce interspecific interactions, were weeded and were watered by automatic sprinklers during the measurement period. The 31 additional species were grown in pots of appropriate sizes (3–10 L, garden soil) in a separate experimental garden ~400 m from the Botanical Garden (see Supplementary Data Table S1 for positions of species in the gardens) and were watered by sprinklers. Both gardens had similar aspect and slope, with the only main difference being more trees in the Botanical Garden. We placed five data loggers measuring soil temperature across these garden parts to obtain information on temperatures over the measurement period. The temperatures of the experimental garden were very similar to mean values in the Botanical Garden, making these two sites easily comparable (see Fig. S1 for temperature course over the measurement period).

The measurement protocol was identical to that used by Mašková *et al.* (2022). In each species, we measured seven shoots (i.e. clearly delimited units with their own connection to the below-ground plant part) biweekly from January until the end of their respective growing season (next January at the latest). We took three measurements of the vegetative parts of above-ground shoots, namely the upper width, lower width, and vegetative length or height (see Mašková *et al.*, 2022 and the figures therein). Yellow senescing leaves were included in the measured parts of the shoot, since they can still photosynthesize and supply nutrients to the rest of the plant. Brown leaves were not included so size parameters generally decreased in the late part of the season. We used these values to calculate the volume occupied by the vegetative part of the shoot and in this way we avoided possible artefacts introduced by only measuring width or height or their simple product. In all analyses, we worked with the cube root of this variable, and we refer to the value as plant size throughout the text (see Supplementary Data Fig. S2 for an example of plant size trajectories).

### Data fitting and extraction of phenological variables

For each measured individual separately, we identified the growing and senescing part of its overall size trajectory throughout the season. This was done by fitting a cubic smoothing spline on the size data of each shoot, finding its maximum value and dividing the measurement points into increasing and declining parts (for details see Supplementary Data Fig. S2).

As the growth of plant size in spring is known to follow a logistic curve (Sun and Frelich 2011; Huang *et al.* 2018), we fitted a logistic function to the growth data using a simplified general logistic function (eqn 1):


$$\begin{array}{l} x=A+\frac{K-}{\left(1+e^{-b*\left(t-a\right)}\right)} \end{array}$$

where *A* is the projected initial size of the individual, *K* is the projected final size of the individual, *a* is the day of maximum growth (inflection point of the logistic curve) and *b* is the standardized growth rate at *a*. We tested fitting models both with non-zero initial size (*A*) and with *A* set to zero. We fitted the logistic function to all values of all individuals of one species together by non-linear mixed model regression using the function nlme from the nlme package in R (R Core Team, 2020). All models had *K* as a random factor at the level of individuals, as individuals typically reached different final sizes. Then we also tested whether allowing the individuals to vary in the remaining parameters increased the model fit; we did this by adding one parameter (*A*, *a* or *b*) at a time as a random factor at the level of individuals to avoid running out of degrees of freedom. In case of no convergence, we sampled the space of initial parameter values until convergence at meaningful parameter values was reached. We chose the model with the lowest Akaike information criterion (AIC) and extracted parameter values (or parameter means if allowed to differ among individuals) of the date of the peak growth (*a*) and standardized growth rate (*b*) for all the species. The initial size was not included in further analyses as it was zero for many species and non-zero values were, in most cases, negligible. The standardized growth rate was log-transformed for all analyses as the generating process is multiplicative. We also calculated the date when the logistic curve reached 25 % of the final size (season start); 25 % was chosen for practical reasons, as smaller percentages of size in spring are generally more variable and prone to be influenced by measurement and fitting artefacts.

To extract parameters of the senescing part of the size trajectory, we followed the approach of Mašková *et al.* (2022). We approximated the shape of the senescence trajectory of each individual separately by means of a spline function fitted to each individual separately using the function smooth.spline with the spar parameter set to 0.5. These splines were averaged and used to obtain approximate dates at which the average individual reached specific fractions of the maximum size of the given species. Following Baudisch *et al.* (2013) and Mašková *et al.* (2022), we extracted three parameters from these predicted values: (1) senescence date, (2) senescence pace and (3) senescence shape (see Table 1 for definitions). Further, we extracted the season end as the date of 25 % of the remaining non-senescent living plant size from the averaged spline curves.

**Table 1.** Definitions of species-specific phenological parameters.

Phenological parameter	Definition
Date of peak growth	Day of the inflexion point of the logistic (the day with the maximum growth rate).
Standardized growth rate	*b* parameter of the logistic (4*d*/*K*), where *d* is the derivative of the logistic at the inflexion point and *K* is the maximum size (asymptote of the logistic).
Senescence date	The first day in the season in which the smoothed size of the declining part reached 50 % of the maximum size of the species.
Senescence pace	The inverse of the number of days (i.e. with units d^−1^) elapsed between the last predicted value being equal to 95 % of the maximum size and the first predicted value being equal to 5 % of the maximum size.
Senescence shape	log(C/D), where C is the difference between the dates on which the predicted value reached 50 % and 5 % of the maximum size of the species, and D is the difference between the dates on which the predicted value reached 50 % and 95 % of the maximum size of the species. Positive values of senescence shape imply an increasing rate of senescence over time.
Season start	The first day in the season in which the logistic curve fitted to the increasing part of the trajectory reached 25 % of the maximum size of the species.
Season end	The first day in the season in which the smoothed size of the declining part of the trajectory reached 25 % of the maximum size of the species.
Season length	Number of days between season start and season end.

Finally, we calculated season length as the number of days between the season start, determined from the logistic function, and the season end, determined from the declining part of the trajectory.

### Additional data on individual species

Trait data for individual species were taken from the following sources: lateral spread from CLOPLA v.3.4 (Klimešová *et al.*, 2017), plant height at maturity from the Pladias database (Chytrý *et al.*, 2021), and SLA and LDMC from the LEDA database (Kleyer *et al.*, 2008), complemented by additional data from the Pladias database (Chytrý *et al.*, 2021). As the CLOPLA database provides data on lateral spread only for clonal plants, an arbitrary value of 0.5 cm per year was imputed for all non-clonal species. In species with no height at maturity available in the Pladias database, we used maximum plant size (*K*) from the fits of the logistic function (eqn 1) on measured growth data as the best estimate of plant height instead.

Niche variables on individual species were taken from two sources: Ellenberg indicator values were taken from Ellenberg *et al.* (1992) and disturbance indicator values from Herben *et al.* (2016). Ellenberg indicator values are known to provide reliable information on species niche along a number of key environmental gradients (Schaffers and Sýkora, 2000; Diekmann, 2003; Wagner *et al.*, 2007; Herben *et al.*, 2018) and possess the desirable property that they characterize the whole species niche, which cannot be easily estimated by direct measurements of field conditions (Schaffers & Sýkora, 2000). Phylogenetic data were taken from the Pladias database (Chytrý *et al.*, 2021).

### Data analysis

The phylogenetic signal in phenological parameters was assessed by the lambda transformation of the inner distances of the phylogenetic tree. The phylogenetic signal was estimated by maximum likelihood estimation using the function pgls from the package caper (v.1.0.1, Orme *et al.*, 2018). As lambda estimates were close to zero for all response variables (Supplementary Data Table S2), we used only non-phylogenetic analyses.

Correlations among the phenological variables were visualized using principal component analysis (PCA) performed in Canoco (v.5.15, ter Braak and Šmilauer, 2012) on centred and standardized data with season length added as a supplementary variable passively projected in the ordination. Eight species were not used in the PCA due to missing values for the end of the season.

We worked with two sets of predictors of season length. The first included functional traits, namely maximum height, SLA, LDMC and lateral spread of clonal plants. Lateral spreading distance and plant height were log-transformed before the analysis to achieve a symmetric distribution. The second set of predictors involved niche (environmental) predictors, namely Ellenberg indicator values for moisture, nutrients, soil reaction, light and temperature, and indicator values for frequency and severity of disturbance.

To examine the effects of these two predictor sets on season length, we proceeded as follows: we first fitted a full model with the whole set of predictors (either all trait predictors or all niche predictors) and iteratively deleted individual model terms using the dredge function from the MuMIn package (v.1.43.17, Bartoń, 2020). Individual models were compared with the best model using the corrected AIC (AICc). The relative importance of each niche predictor was estimated by the sum of the AICc weights of the models in which the given predictor appeared, divided by the sum of the AICc weights of all models, taking into account only models that differed from the best model by < 3 Akaike units. The effects of individual predictor terms that appeared at least once in this set of models were assessed by Akaike-weighted means of the estimates of standardized regression coefficients from all models in this set that included that term. The 95 % confidence intervals of the estimates were determined by the same procedure. Responses of the two variables of spring growth (date of peak growth and standardized growth rate) to trait and niche parameters were analysed in the same way as for season length. The corresponding analysis of senescence parameters has been published by Mašková *et al.* (2022).

Finally, we used structural equation modelling to examine how trait and niche variables affect season length via individual phenological variables of both spring and autumn size change. SEMs were fitted using local estimation (Lefcheck, 2016) based on three sets of models: (1) a model of season length as a function of individual phenological variables (date of peak growth, standardized growth rate, senescence date, senescence rate and senescence shape), (2) models of these phenological variables as functions of species functional traits, namely height at maturity, lateral spread, LDMC and SLA, and (3) models of these traits as functions of niche variables (moisture, light, soil reaction and disturbance frequency). Species traits and niche variables used in the analysis were selected based on previous univariate relationships to phenological variables. The combined set of these models was simplified by deleting non-significant predictors, and then testing potentially missing relationships among variables by tests of directed separation. Significant independence claims were used to add regression terms if variables in question were at different levels of the hierarchy (e.g. a phenological variable and a trait variable), or to add correlated error terms for variables at the same hierarchy level (i.e. within phenological variables, and within trait variables). This was repeated until no significant independence claims existed and the significance of Fisher’s C was > 0.2. The difference in AIC values was used as an additional criterion for model building. The SEM was fitted using the package piecewiseSEM (v.2.1.0.; see Lefcheck, 2016) for R.

## RESULTS

The species-specific season length varied substantially from 73 d in *Allium oleraceum* to 337 d in *Geum urbanum*; in eight species the above-ground parts overwintered (i.e. their volume did not decrease below 25 % of the maximum plant size), and they were assigned a season length of 365 d.

**Fig. 1.**
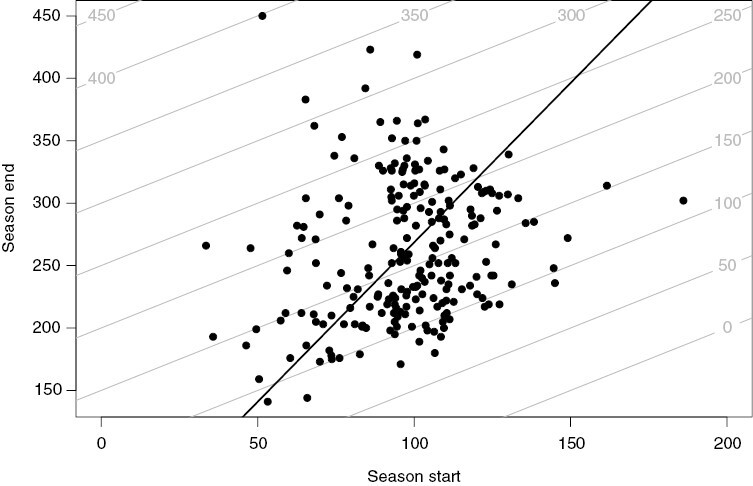
Relationship between the mean dates of 25 % of plant size in growth (Season start) and in senescence phase (Season end). The black line was fitted by standardized major axis regression (R package smatr, *R*^2^ = 0.036, *P* = 0.04). The grey lines are isoclines of equal season length.

**Fig. 2.**
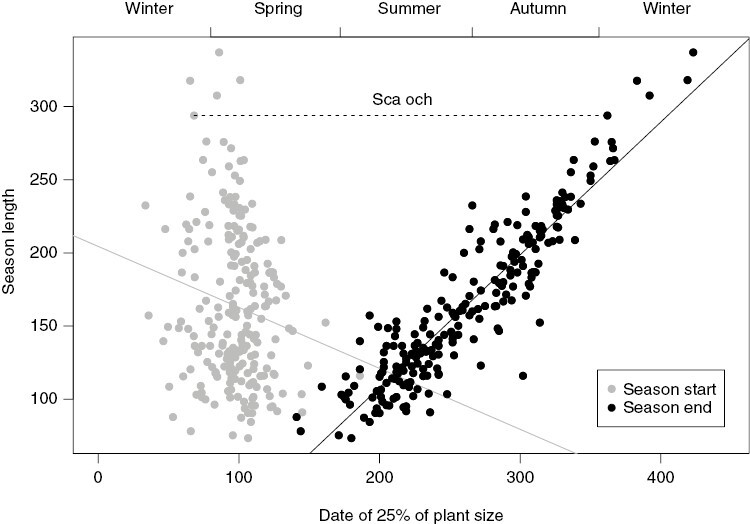
Relationships between season length and its two sources, the mean dates of 25 % of plant size in growth (Season start, grey, adjusted *R*^2^ = 0.02, *P* = 0.012) and in senescence phase (Season end, black, adjusted *R*^2^ = 0.85, *P* < 0.001). The species that did not fall below 25 % of the remaining plant size and were assigned to have their season 365 d long are not plotted or included in the correlations. The dashed line depicts one example of season length in *Scabiosa ochroleuca*.

There was only a weak relationship between the dates of season start and season end (Fig. 1). Interspecific variation in the season end (s.d. = 58.6 d) was more than twice as large compared to interspecific variation in the season start (s.d. = 23.7 d, see also Fig. 2). Consequently, the univariate correlation between the season length and start of the season was loose (Fig. 2, *R*^2^_adj_ = 0.02, *P* = 0.012), while the correlation with the end of the season was much tighter (Fig. 2, *R*^2^_adj_ = 0.85, *P* < 0.001).

**Fig. 3.**
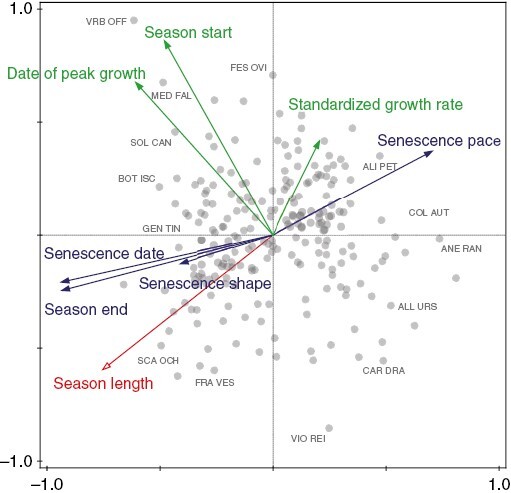
Principal component analysis (PCA) of all growth phenology (green) and senescence phenology (blue) parameters together. We added mean dates of 25 % of plant size for both growing (Season start) and senescence (Season end) phenology since they were used to compute season length. Season length (red) is passively projected in the ordination. Species positions are marked by grey circles with the most extreme ones described by names. ALI PET – *Alliaria petiolata*, ALL URS – *Allium ursinum*, ANE RAN – *Anemone ranunculoides*, BOT ISC – *Bothriochloa ischaemum*, CAR DRA – *Cardaria draba*, COL AUT – *Colchicum autumnale*, FES OVI – *Festuca ovina*, FRA VES – *Fragaria vesca*, GEN TIN – *Genista tinctoria*, MED FAL – *Medicago falcata*, SCA OCH – *Scabiosa ochroleuca*, SOL CAN – *Solidago canadensis*, VIO REI – *Viola reichenbachiana*, VRB OFF – *Verbena officinalis*. *N* = 223 species.

The phenological variables almost entirely predicted season length (Table 2, *R*^2^ = 0.985). Its best predictor was the senescence date (*r* = 0.96) followed by the date of peak growth (*r* = −0.39). The exploratory PCA of all the phenological variables (see Fig. 3 and Supplementary Data Table S3 for correlation matrix of all phenological variables) showed that there were two main axes of variation in the dataset. The first axis (43.7 % of variation explained) was correlated with senescence date, pace and season end, that is it largely captured the pace–date axis of shoot senescence (Mašková *et al.*, 2022). The second axis (23.2 % of variation explained) was correlated with season start, date of peak growth and standardized growth rate. Season length was correlated with the first axis (i.e. with senescence variables, *r* = −0.75) more than with the second axis (i.e. with growth variables, *r* = −0.6). Spring geophytes such as *Anemone ranunculoides* and *Allium ursinum* were placed at the positive end of the first axis as they were characterized, in addition to an early start, by early senescence as opposed to late-senescing species such as the evergreen *Genista tinctoria* or a C4 grass, *Bothriochloa ischaemum*. Some rosette species, for example *Fragaria vesca*, were placed at the negative end of the second axis as they grew slowly but started early in the season and their unfinished growth by the end of the year led to higher season lengths. Finally, late species could have had both a long growing season (as in the case of the late-flowering dry grassland *Scabiosa ochroleuca*), but also a medium length growing season caused by their late start (e.g. the tall *Solidago canadensis*).

**Table 2.** Prediction of season length by phenological variables (*N* = 223, *R*^2^ = 0.985) calculated by multiple regression.

	Estimate	Confidence interval
Date of peak growth	−0.389	−0.407 to −0.372
Standardized growth rate (log)	−0.211	−0.227 to −0.194
Senescence date	0.960	0.935 to 0.985
Senescence pace	−0.142	−0.163 to −0.121
Senescence shape	−0.126	−0.145 to −0.108

**Fig. 4.**
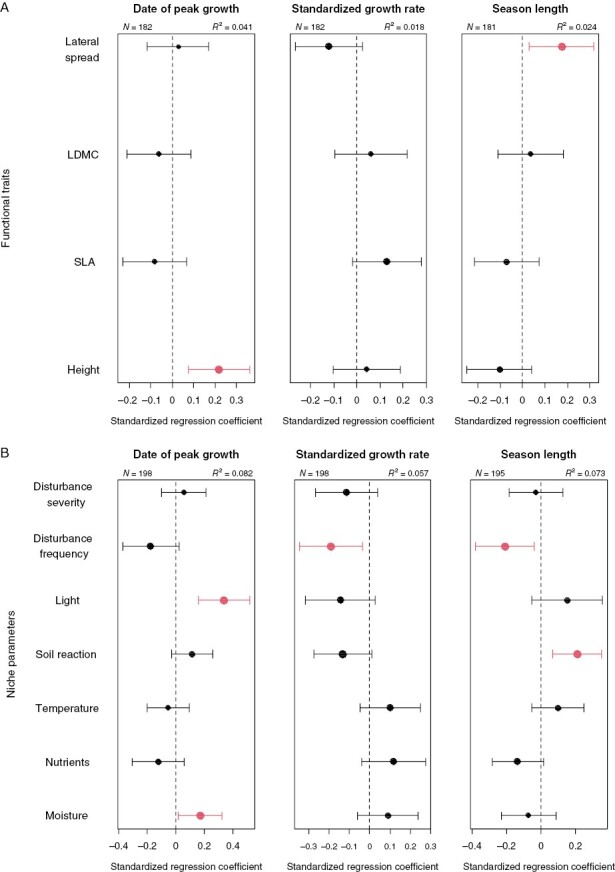
Effects of species traits (A) and niche variables (B) on date of peak growth, standardized growth rate and season length. Points indicate estimates of standardized regression coefficients; error bars, their 95 % confidence intervals. Predictors with confidence intervals not covering zero are shown in red. Point size is proportional to the relative importance of the given predictor (i.e. the weighted occurrence of the predictor in the set of best models). Note the much larger proportion of variance explained by niche variables compared to traits. For similar analyses of the senescence parameters see Mašková *et al.* (2022). For further details, see the Methods section.

In the analysis of the effect of functional traits on season length, only lateral spread (i.e. a measure of plant clonality) was significantly correlated with season length (Fig. 4A). Those species with longer spacers (rhizomes, stolons) had longer growing seasons than species with short (e.g. bulbs and tubers) or no clonal growth. The date of peak growth was later in taller plants, while no traits correlated with standardized growth rate. Season length was predicted by two variables describing species niche (Fig. 4B), namely disturbance frequency and soil reaction, while the effect of nutrients was only marginal. The species from calcium-rich habitats with higher pH and infrequent disturbance had longer season length. The date of peak growth was correlated with high moisture and light, while the standardized growth rate was high in species from habitats with low disturbance frequency.

**Fig. 5.**
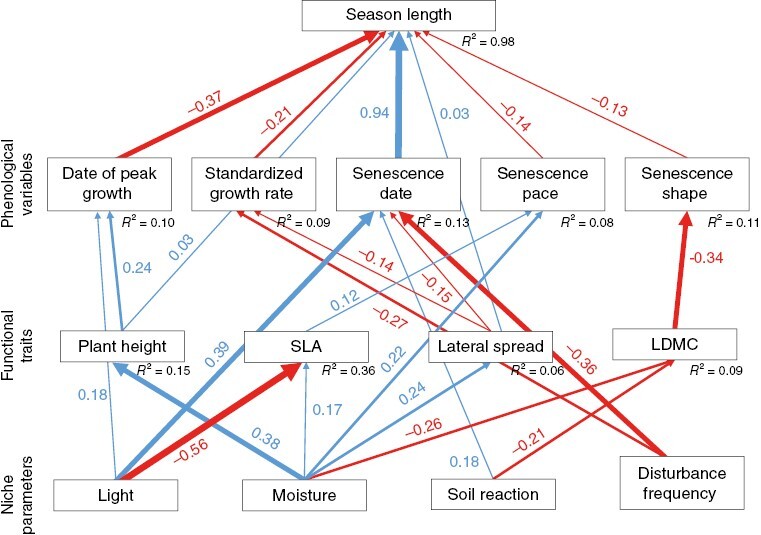
Path diagram of the model of season length. Blue arrows indicate positive relationships, red arrows negative relationships; strength of the relationship is indicated by line thickness and by standardized partial regression coefficients associated with individual arrows. A positive senescence shape means increasing rate of senescence with time. Fisher’s *C* = 107.0 with *P* = 0.455 and 106 degrees of freedom. The following correlated errors are included in the model (not shown in the figure): Date of peak growth – Senescence date (*r* = 0.32), Date of peak growth – Standardized growth rate (*r* = −0.21), Senescence date – Standardized growth rate (*r* = −0.20), Senescence date – Senescence pace (*r* = −0.57), Senescence date – Senescence shape (*r* = 0.33), Date of peak growth - Senescence pace (*r* = −0.16), Standardized growth rate - Senescence pace (*r* = 0.17), and LDMC – SLA (*r* = −0.36).

The SEM (Fig. 5) showed that while the niche variables had a large effect on all examined traits, these relationships only weakly translated into effects on phenological variables and hence season length. Among the plant traits, height, lateral spread and LDMC had stronger effects on phenological variables, while the effect of SLA was only small. Importantly, several trait variables had opposing effects on season length through spring and autumn variables. Plant height had a negative effect on season length through its effect on date of peak growth, but also a direct positive effect. Similarly, lateral spread had a positive effect on season length both through standardized growth rate and directly, but had a negative effect through senescence date.

Habitat factors generally had stronger direct effects compared to traits. There was a strong direct positive effect of light on the senescence date and through it on season length, which was partly reduced by the direct effect of light on the date of peak growth. This means that plants of open habitats (high light) have longer seasons that are shifted to later dates. The strong negative effect of light on SLA did not greatly influence season length because the effect of SLA on phenological variables was minor, only slightly decreasing the senescence pace in plants from open habitats. Moisture affected most traits and phenological variables. It had a negative effect on season length through the late growth start of tall plants that were more abundant in wet habitats (high moisture). The positive effect of moisture on lateral spread resulted in a negative effect on season length because more clonally propagating plants abundant in wet habitats generally have later senescence dates. Soil reaction had a direct positive effect on senescence date leading to a positive effect on season length, but it was diminished by the effect of soil reaction on LDMC and its negative effect on season length. This means that plants from alkaline soils have less durable leaves (low LDMC), which have a more positive (accelerating) shape of senescence. Finally, disturbance frequency had a negative effect on senescence date, causing a strong negative effect on season length, but this was again partly reduced by the negative correlation with the standardized growth rate.

## DISCUSSION

### Components of season length

Our analysis of a large set of perennial species showed that interspecific differences in season length were determined primarily by the season end and associated senescence variables, while season start was a relatively minor predictor. This is obviously caused by the fairly synchronous spring growth, which is driven by both risk avoidance in early spring and subsequent fast growth and competition for light (Huang *et al.*, 2019). In contrast, senescence in some species starts as early as late spring while in others it extends until winter, making its distribution markedly bimodal (Mašková *et al.*, 2022). Some species are damaged by unpredictable summer droughts or early frost leading to early senescence both in mid-summer and in late autumn, while some other species show developmental senescence early in the season. In contrast, other plants extend their season more into a later phase as the loss of above-ground organs then amounts only to loss of part of their resources. Consequently, they can afford the risk of frost damage in exchange for more time to store valuable resources in below-ground storage organs (May and Killingbeck, 1992) or invest in better frost resistance (Bucher *et al.*, 2019). Although there is much less solar radiation in autumn than in spring, some species can still acquire a significant part of their carbon budget late in the season, at least in forest herbs (Heberling *et al.*, 2019). The major effect of autumn variables on season length underlies essentially no phylogenetic pattern in season length, in spite of a fairly strong phylogenetic signal in spring growth (Huang *et al.*, 2018). Senescence phenology is known to bear almost no phylogenetic signal (Mašková *et al.*, 2022); the only trait with phylogenetic signal, namely senescence shape, had only a small effect on season length.

Our definitions of the season start and end and the five phenological variables underlie the almost exact prediction of season length by phenological variables. While the prime determinants of season length were dates of peak growth and senescence, both spring growth rate and senescence pace also contributed. The effect of both of them is negative as low growth rate and/or slow senescence pace extend the period over which the plant changes its volume in both directions and thus shift the beginning and end of the season toward extreme dates (i.e. early spring or late autumn).

### Determinants of season length

The start and the end of the season were more or less independent of each other, which is similar to a result found in a global dataset of trees (Panchen *et al.*, 2015). This contrasts with an analysis of a set of tree species and from satellite data (Keenan and Richardson, 2015) where spring temperatures influenced senescence dates. While their results are not directly comparable with ours, as their measure of spring start is usually leaf-out with different correlation with temperature compared to our growth-based estimate, it is unlikely that both these components would in principle be uncorrelated even in herbs.

A closer look at the determinants of both spring and autumn phenological variables shows that they are at last partly driven by the same traits and respond to the same environmental pressures. The SEM has shown that the niche variables (mainly light, moisture and disturbance frequency) and some traits (height and lateral spread) affected the phenological variables and thus account for their interrelationships. However, their effects on season length through spring and autumn phenological variables often cancelled each other. In particular, the niche variables often had contrasting effects influencing season length differently through different traits and phenological variables.

Major trait determinants of season length are plant height and lateral spread. Tall plants generally grow somewhat later in the season as getting tall takes time; they flower later and are therefore forced to extend their senescence until winter to store resources. The longer season in clonal plants can be attributed to the extra time and investment it takes to build spacers and new ramets (Stuefer *et al.*, 2002; Martínková *et al.*, 2020), and it is, in fact, analogous to height, but in a horizontal direction. Both parameters of the leaf economy spectrum had distinct albeit small effects: SLA was related to faster growth and faster senescence, while LDMC influenced only senescence shape (negatively).

All the examined species traits were, as expected, associated with differences in species niches, but only some of these relationships had cascading effects on the phenological variables. Additionally, several niche variables influenced the phenological variables directly. This might have been caused simply by the limited set of traits we used. We might have omitted some other traits which would be better predictors of either spring or autumn phenology, but recent publications searching for trait predictors of interspecific differences of species phenology actually use similar traits to us (König *et al.*, 2017; Sporbert *et al.*, 2022). One of the reasons might also be that each niche variable is likely to select several different species strategies which cannot be simply captured by mean trait values (Castillioni *et al.*, 2022).

In many cases, individual predictors had opposing effects on season length. For example, tall plants had the peak of their growth delayed in spring but also had generally later season ends, meaning they had their vegetative season shifted more towards the end of the season. Similar effects are found in niche parameters. Low disturbance frequency (i.e. primarily forest habitats) selected both species with high spring growth rate and species with late senescence, which can be attributed to the two main strategies of forest species, namely early spring geophytes and shade-tolerant late species. Finally, species of wet habitats started their growth later and senesced faster leading to shorter season lengths, but also included clonal species, which have longer season length. This shows that neither traits nor niche variables can explain the differences in season lengths on their own, and we have to look closer to disentangle various strategies of plants from each other.

## CONCLUSIONS

The study makes clear that interspecific differences in season length are due primarily to the processes in autumn. The large differences among species in spring and autumn determinants of season length indicate very different drivers and potential risks in growth and senescence. While interspecific differences in spring and autumn phenology may be correlated with similar plant functional traits, the role of these traits in tradeoffs that the plants face in their photosynthetic and nutrient economies is bound to be different in senescence and growth (Sporbert *et al.*, 2022). The SEM also shows that the key to understanding spring growth, senescence and hence overall season length lies in differences in the environment where these species typically occur (their niches) and are not easily captured by trait differences in these species (with the exception of plant height and, to a lesser extent, lateral spread).

Current global change is affecting the phenology of plants (Peñuelas *et al.*, 2002; Parmesan and Yohe, 2003; Menzel *et al.*, 2006), leading to season shifts and changes in season length (Linderholm, 2006; Cleland *et al.*, 2007; Piao *et al.*, 2007). The simultaneity of spring growth indicates that season length could be far more affected by changes in temperatures and precipitation in early spring than at the end of the growing season because of the large temporal spread of season end dates. Further, the predicted changes in the quantity and timing of summer precipitation (Ummenhofer and Meehl, 2017) might cause changes in the first wave of senescence because of direct effects of drought. More detailed knowledge on specific drivers of timing of spring and autumn phenological events is therefore indispensable (Nordt *et al.*, 2021). Identification of all these effects should be based on extensive time series of temperature, precipitation and light measurements collected alongside phenological measurements to determine how temperature and day length signals combine with direct effects of drought and frost. The fairly strong role of niche-based predictors found in this study, namely of water and light availability, implies that the shifts in phenological events and species-specific season length due to global change are probably not uniform across the whole flora and their interactions with light and moisture gradients should be taken into account.

## SUPPLEMENTARY DATA

Supplementary data are available online at https://academic.oup.com/aob and consist of the following.

Table S1: Details of all used species and the garden parts where they grew. Table S2: The results of phylogenetic analysis of the phenological variables. Table S3: Correlations between the phenological variables. Fig. S1: The course of daily mean soil temperatures in four places in the botanical garden and in the experimental garden. Fig. S2: Definition of the growth and senescence parts of the size trajectory and an example of individual trajectories for one species with season start and end marked.

mcad088_suppl_Supplementary_Material
