# Supplementary material for: Determinants of interspecific variation in season length of perennial herbs
Source: Ann Bot. 2023 Jul 3;132(2):281–91. doi: 10.1093/aob/mcad088 (PMC10583191; doi:10.1093/aob/mcad088)
Supplement: mcad088_suppl_Supplementary_Material [file mcad088_suppl_supplementary_material.docx]

## Supplementary material

Article title: Determinants of interspecific variation in season length of perennial herbs

Authors: Tomáš Koubek, Tereza Mašková, Tomáš Herben

The following Supplementary material is available for this article:

**Table S1** Table of all used species and the garden parts where they grew.

**Table S2** The results of the phylogenetic analysis of the phenological variables.

**Table S3** Correlations between the phenological variables.

**Fig. S1** The course of daily mean soil temperatures in four places in the botanical garden and in the experimental garden.

**Fig. S2** The definition of the growth and senescence parts of the size trajectory and an example of individual trajectories for one species with season start and end marked.

**Table S1** Table of all used species and the garden parts where they grew.

| species | garden part |
| --- | --- |
| Agropyron pectinatum | genetic garden (in pots) |
| Achillea collina | genetic garden (in pots) |
| Achillea millefolium | genetic garden (in pots) |
| Arrhenatherum elatius | genetic garden (in pots) |
| Calamagrostis arundinacea | genetic garden (in pots) |
| Calamagrostis epigejos | genetic garden (in pots) |
| Cardaria draba | genetic garden (in pots) |
| Cirsium canum | genetic garden (in pots) |
| Cruciata glabra | genetic garden (in pots) |
| Galega officinalis | genetic garden (in pots) |
| Galium pumilum | genetic garden (in pots) |
| Helianthus tuberosus | genetic garden (in pots) |
| Hieracium murorum | genetic garden (in pots) |
| Inula oculus-christi | genetic garden (in pots) |
| Isatis tinctoria | genetic garden (in pots) |
| Lotus tenuis | genetic garden (in pots) |
| Medicago falcata | genetic garden (in pots) |
| Mentha longifolia | genetic garden (in pots) |
| Prunella laciniata | genetic garden (in pots) |
| Rumex obtusifolius | genetic garden (in pots) |
| Rumex sanguineus | genetic garden (in pots) |
| Sanicula europea | genetic garden (in pots) |
| Scabiosa columbaria | genetic garden (in pots) |
| Scorzonera hispanica | genetic garden (in pots) |
| Senecio ovatus | genetic garden (in pots) |
| Seseli osseum | genetic garden (in pots) |
| Solidago canadensis | genetic garden (in pots) |
| Solidago virgaurea | genetic garden (in pots) |
| Tanacetum corymbosum | genetic garden (in pots) |
| Valeriana officinalis | genetic garden (in pots) |
| Viola collina | genetic garden (in pots) |
| Aconitum lycoctonum | medicinal plants (cultivated) |
| Achillea ptarmica | medicinal plants (cultivated) |
| Alisma plantago-aquatica | medicinal plants (cultivated) |
| Bistorta major | medicinal plants (cultivated) |
| Caltha palustris | medicinal plants (cultivated) |
| Comarum palustre | medicinal plants (cultivated) |
| Convallaria majalis | medicinal plants (cultivated) |
| Genista tinctoria | medicinal plants (cultivated) |
| Glyceria maxima | medicinal plants (cultivated) |
| Lamium album | medicinal plants (cultivated) |
| Lycopus europaeus | medicinal plants (cultivated) |
| Lysimachia nummularia | medicinal plants (cultivated) |
| Nepeta cataria | medicinal plants (cultivated) |
| Ononis spinosa | medicinal plants (cultivated) |
| Origanum vulgare | medicinal plants (cultivated) |
| Parietaria officinalis | medicinal plants (cultivated) |
| Potentilla erecta | medicinal plants (cultivated) |
| Potentilla reptans | medicinal plants (cultivated) |
| Prunella vulgaris | medicinal plants (cultivated) |
| Sanguisorba officinalis | medicinal plants (cultivated) |
| Saponaria officinalis | medicinal plants (cultivated) |
| Schoenoplectus tabernaemontani | medicinal plants (cultivated) |
| Verbena officinalis | medicinal plants (cultivated) |
| Veronica officinalis | medicinal plants (cultivated) |
| Agrimonia eupatoria | rocky and dry grassland |
| Achillea nobilis | rocky and dry grassland |
| Achillea pannonica | rocky and dry grassland |
| Allium senescens ssp. montanum | rocky and dry grassland |
| Alopecurus pratensis | rocky and dry grassland |
| Anemone sylvestris | rocky and dry grassland |
| Anthericum liliago | rocky and dry grassland |
| Arenaria grandiflora | rocky and dry grassland |
| Artemisia pontica | rocky and dry grassland |
| Asperula tinctoria | rocky and dry grassland |
| Aster amellus | rocky and dry grassland |
| Astragalus glycyphyllos | rocky and dry grassland |
| Aurinia saxatilis | rocky and dry grassland |
| Bothriochloa ischaemum | rocky and dry grassland |
| Briza media | rocky and dry grassland |
| Bromus benekenii | rocky and dry grassland |
| Bromus erectus | rocky and dry grassland |
| Bromus inermis | rocky and dry grassland |
| Carex digitata | rocky and dry grassland |
| Carex muricata | rocky and dry grassland |
| Carex pilosa | rocky and dry grassland |
| Carex tomentosa | rocky and dry grassland |
| Carex vulpina | rocky and dry grassland |
| Cirsium acaulon | rocky and dry grassland |
| Clematis integrifolia | rocky and dry grassland |
| Clematis recta | rocky and dry grassland |
| Colchicum autumnale | rocky and dry grassland |
| Coronilla vaginalis | rocky and dry grassland |
| Cynosurus cristatus | rocky and dry grassland |
| Deschampsia cespitosa | rocky and dry grassland |
| Dianthus armeria | rocky and dry grassland |
| Dianthus carthusianorum | rocky and dry grassland |
| Dianthus gratianopolitanus | rocky and dry grassland |
| Digitalis grandiflora | rocky and dry grassland |
| Echium vulgare | rocky and dry grassland |
| Elymus hispidus | rocky and dry grassland |
| Euphorbia cyparissias | rocky and dry grassland |
| Festuca gigantea | rocky and dry grassland |
| Festuca ovina | rocky and dry grassland |
| Festuca pallens | rocky and dry grassland |
| Festuca pratensis | rocky and dry grassland |
| Festuca rupicola | rocky and dry grassland |
| Festuca valesiaca | rocky and dry grassland |
| Filipendula vulgaris | rocky and dry grassland |
| Fragaria vesca | rocky and dry grassland |
| Fragaria viridis | rocky and dry grassland |
| Galium boreale | rocky and dry grassland |
| Geranium pratense | rocky and dry grassland |
| Geranium sanguineum | rocky and dry grassland |
| Geranium sylvaticum | rocky and dry grassland |
| Helianthemum grandiflorum subsp. obscurum | rocky and dry grassland |
| Helictochloa pratensis | rocky and dry grassland |
| Hepatica nobilis | rocky and dry grassland |
| Hierochloë australis | rocky and dry grassland |
| Hypericum maculatum | rocky and dry grassland |
| Inula britannica | rocky and dry grassland |
| Inula ensifolia | rocky and dry grassland |
| Inula hirta | rocky and dry grassland |
| Inula salicina | rocky and dry grassland |
| Iris graminea | rocky and dry grassland |
| Lathyrus niger | rocky and dry grassland |
| Leontodon hispidus | rocky and dry grassland |
| Linaria vulgaris | rocky and dry grassland |
| Linum flavum | rocky and dry grassland |
| Lithospermum officinale | rocky and dry grassland |
| Lithospermum purpurocaeruleum | rocky and dry grassland |
| Lotus corniculatus | rocky and dry grassland |
| Lythrum virgatum | rocky and dry grassland |
| Maianthemum bifolium | rocky and dry grassland |
| Melica uniflora | rocky and dry grassland |
| Molinia caerulea | rocky and dry grassland |
| Peucedanum cervaria | rocky and dry grassland |
| Phleum phleoides | rocky and dry grassland |
| Phleum pratense | rocky and dry grassland |
| Phlomis tuberosa | rocky and dry grassland |
| Poa compressa | rocky and dry grassland |
| Potentilla argentea | rocky and dry grassland |
| Potentilla heptaphylla | rocky and dry grassland |
| Potentilla incana | rocky and dry grassland |
| Potentilla recta | rocky and dry grassland |
| Primula veris | rocky and dry grassland |
| Pulsatilla pratensis subsp. bohemica | rocky and dry grassland |
| Ranunculus acris | rocky and dry grassland |
| Salvia glutinosa | rocky and dry grassland |
| Salvia nemorosa | rocky and dry grassland |
| Sanguisorba minor | rocky and dry grassland |
| Scabiosa ochroleuca | rocky and dry grassland |
| Sesleria caerulea | rocky and dry grassland |
| Silene latifolia subsp. alba | rocky and dry grassland |
| Silene vulgaris | rocky and dry grassland |
| Stachys recta | rocky and dry grassland |
| Stellaria holostea | rocky and dry grassland |
| Stipa capillata | rocky and dry grassland |
| Stipa tirsa | rocky and dry grassland |
| Succisa pratensis | rocky and dry grassland |
| Teucrium chamaedrys | rocky and dry grassland |
| Thalictrum minus | rocky and dry grassland |
| Trifolium medium | rocky and dry grassland |
| Trifolium pratense | rocky and dry grassland |
| Trollius altissimus | rocky and dry grassland |
| Veronica prostrata | rocky and dry grassland |
| Veronica spicata | rocky and dry grassland |
| Veronica teucrium | rocky and dry grassland |
| Vicia cassubica | rocky and dry grassland |
| Vincetoxicum hirundinaria | rocky and dry grassland |
| Viola reichenbachiana | rocky and dry grassland |
| Viscaria vulgaris | rocky and dry grassland |
| Anchusa officinalis | sandy open grassland |
| Armeria vulgaris | sandy open grassland |
| Calamagrostis villosa | sandy open grassland |
| Corynephorus canescens | sandy open grassland |
| Juncus effusus | sandy open grassland |
| Juncus filiformis | sandy open grassland |
| Juncus inflexus | sandy open grassland |
| Koeleria macrantha | sandy open grassland |
| Polemonium caeruleum | sandy open grassland |
| Silene nutans | sandy open grassland |
| Acorus calamus | under trees and by the stream |
| Actaea spicata | under trees and by the stream |
| Adoxa moschatellina | under trees and by the stream |
| Alchemilla monticola | under trees and by the stream |
| Alliaria petiolata | under trees and by the stream |
| Allium oleraceum | under trees and by the stream |
| Allium ursinum | under trees and by the stream |
| Anemone ranunculoides | under trees and by the stream |
| Anthoxanthum odoratum | under trees and by the stream |
| Aquilegia vulgaris | under trees and by the stream |
| Aristolochia clematitis | under trees and by the stream |
| Betonica officinalis | under trees and by the stream |
| Brachypodium sylvaticum | under trees and by the stream |
| Carex brizoides | under trees and by the stream |
| Carex flacca | under trees and by the stream |
| Carex hirta | under trees and by the stream |
| Carex leporina | under trees and by the stream |
| Carex melanostachya | under trees and by the stream |
| Carex remota | under trees and by the stream |
| Circaea lutetiana | under trees and by the stream |
| Cirsium oleraceum | under trees and by the stream |
| Cladium mariscus | under trees and by the stream |
| Dactylis glomerata | under trees and by the stream |
| Dentaria bulbifera | under trees and by the stream |
| Epilobium hirsutum | under trees and by the stream |
| Festuca arundinacea | under trees and by the stream |
| Festuca heterophylla | under trees and by the stream |
| Festuca rubra | under trees and by the stream |
| Filipendula ulmaria | under trees and by the stream |
| Fragaria moschata | under trees and by the stream |
| Galium odoratum | under trees and by the stream |
| Geranium palustre | under trees and by the stream |
| Geum urbanum | under trees and by the stream |
| Hacquetia epipactis | under trees and by the stream |
| Hieracium sabaudum | under trees and by the stream |
| Holcus lanatus | under trees and by the stream |
| Chaerophyllum aromaticum | under trees and by the stream |
| Iris sibirica | under trees and by the stream |
| Iris spuria | under trees and by the stream |
| Lathyrus transsilvanicus | under trees and by the stream |
| Lathyrus vernus | under trees and by the stream |
| Leonurus cardiaca | under trees and by the stream |
| Leucojum vernum | under trees and by the stream |
| Lysimachia punctata | under trees and by the stream |
| Lythrum salicaria | under trees and by the stream |
| Petasites albus | under trees and by the stream |
| Plantago media | under trees and by the stream |
| Poa nemoralis | under trees and by the stream |
| Potentilla alba | under trees and by the stream |
| Rumex thyrsiflorus | under trees and by the stream |
| Scrophularia nodosa | under trees and by the stream |
| Scutellaria altissima | under trees and by the stream |
| Sesleria uliginosa | under trees and by the stream |
| Stachys palustris | under trees and by the stream |
| Stachys sylvatica | under trees and by the stream |
| Symphytum cordatum | under trees and by the stream |
| Symphytum officinale | under trees and by the stream |
| Veronica chamaedrys | under trees and by the stream |
| Vinca minor | under trees and by the stream |

**Table S2** The results of the phylogenetic analysis of the phenological variables. As most values of lambda are low, we used non-phylogenetic analyses throughout the paper.

|  | lambda | Lower 95% CI | Upper 95% CI | p (different from zero) | p (different from one) | N |
| --- | --- | --- | --- | --- | --- | --- |
| Season length | 0.079 | NA | 0.364 | 0.0745 | 0 | 225 |
| Date of peak growth | 0.102 | 0.018 | 0.294 | 0.003 | 0 | 228 |
| Standardized growth rate (log) | 0.072 | NA | 0.266 | 0.1986 | 0 | 228 |
| Senescence date | 0.132 | 0.024 | 0.405 | 0.0013 | 0 | 227 |
| Senescence pace | 0 | NA | 0.270 | 1 | 0 | 228 |
| Senescence shape | 0.216 | 0.068 | 0.455 | 0 | 0 | 220 |

**Table S3** Correlations between the phenological variables. Positive values are in green, negative values in red.

|  | Season length | Season start | Season end | Date of peak growth | Stand. growth rate | Sen. date | Sen. pace | Sen. shape |
| --- | --- | --- | --- | --- | --- | --- | --- | --- |
| Season length |  | -0.168 | 0.920 | 0.026 | -0.324 | 0.890 | -0.700 | 0.281 |
| Season start | -0.168 |  | 0.232 | 0.840 | 0.268 | 0.264 | -0.042 | 0.096 |
| Season end | 0.920 | 0.232 |  | 0.360 | -0.214 | 0.983 | -0.707 | 0.315 |
| Date of peak growth | 0.026 | 0.840 | 0.360 |  | -0.159 | 0.370 | -0.169 | 0.029 |
| Stand. growth rate | -0.324 | 0.268 | -0.214 | -0.159 |  | -0.191 | 0.155 | 0.081 |
| Sen. date | 0.890 | 0.264 | 0.983 | 0.370 | -0.191 |  | -0.622 | 0.441 |
| Sen. pace | -0.700 | -0.042 | -0.707 | -0.169 | 0.155 | -0.622 |  | -0.126 |
| Sen. shape | 0.281 | 0.096 | 0.315 | 0.029 | 0.081 | 0.441 | -0.126 |  |

**Fig. S1** The course of daily mean soil temperatures (5 cm deep) in the four places in botanical garden (we did not have a data logger by the medicinal plants) and in the experimental garden.

|  |
| --- |
| 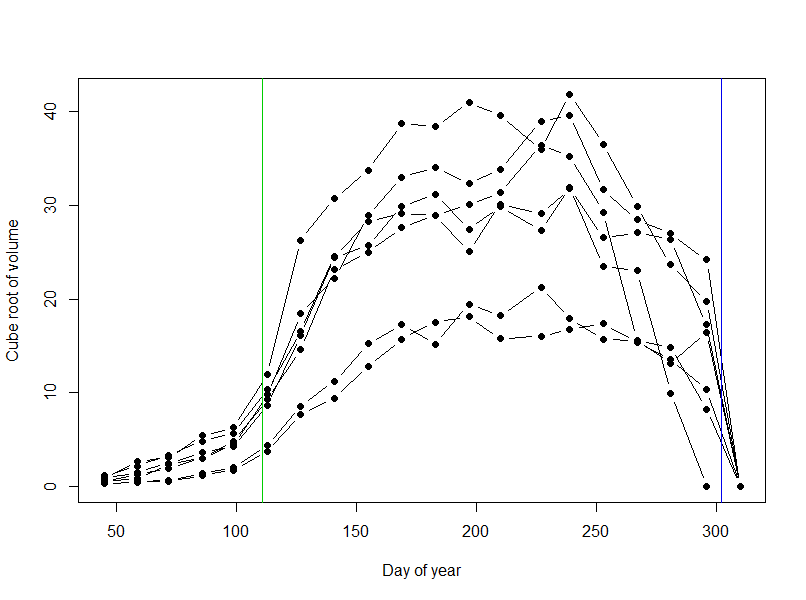 |
|  |

**Fig. S2 Upper pane:** definition of the growth and senescence parts of the size trajectory. This was done by fitting a cubic smoothing spline on the size data of each individual. We used the function smooth.spline, with a dynamically determined number of knots based on the data set size (R Core Team 2020). We used the parameter 'spar' to control the degree of smoothing (to set the coefficient λ of the integral of the squared second derivative in the fit (penalized log likelihood) by setting it to 0.6. To determine the growing part of the growth trajectory, we found the maximum of the smoothed growth curve (in no case were there two maxima) and retained all points to the left of the maximum (i.e. the increasing part of the spline); to the right, we retained all contiguous points lying above the spline directly adjacent to the maximum and discarded all points to the right of it, beginning with the first point lying below the spline. Similarly, to the growing part, we identified the senescing part of the overall trajectory of size throughout the season. To determine the senescing part of the growth trajectory, we found the maximum of the spline curve and retained all points to the right of the maximum (i.e. the declining part of the spline); to the left, we retained all contiguous points lying above the spline directly adjacent to the maximum and discarded all points to the left of it, beginning with the first point lying below the spline. Note that one point is part of both senescing and growth parts of the growth trajectory (by definitions used, the number of such shared points may vary from zero to several.) **Lower pane:** Example of individual growth trajectories for *Agrimonia eupatoria*. Each line corresponds to one individual (shoot). The green line marks the computed season start for the given species; blue line marks the season end. For calculation of these dates see the Methods section.
